# Mechanism of mesenchymal stem cells in spinal cord injury repair through macrophage polarization

**DOI:** 10.1186/s13578-021-00554-z

**Published:** 2021-02-23

**Authors:** Nan An, Jiaxu Yang, Hequn Wang, Shengfeng Sun, Hao Wu, Lisha Li, Meiying Li

**Affiliations:** 1 The Key Laboratory of Pathobiology, Ministry of Education, Jilin University, 126 Xinmin Street, Changchun, 130021 Jilin People’s Republic of China; 2 The Second Hospital of Jilin University, Changchun, 130021 Jilin China; 3 The First Hospital of Jilin University, Changchun, 130021 Jilin China

**Keywords:** Spinal cord injury, Inflammatory microenvironment, Macrophage polarization, Mesenchymal stem cells

## Abstract

Treatment and rehabilitation of spinal cord injury (SCI) is a major problem in clinical medicine. Modern medicine has achieved minimal progress in improving the functions of injured nerves in patients with SCI, mainly due to the complex pathophysiological changes that present after injury. Inflammatory reactions occurring after SCI are related to various functions of immune cells over time at different injury sites. Macrophages are important mediators of inflammatory reactions and are divided into two different subtypes (M1 and M2), which play important roles at different times after SCI. Mesenchymal stem cells (MSCs) are characterized by multi-differentiation and immunoregulatory potentials, and different treatments can have different effects on macrophage polarization. MSC transplantation has become a promising method for eliminating nerve injury caused by SCI and can help repair injured nerve tissues. Therapeutic effects are related to the induced formation of specific immune microenvironments, caused by influencing macrophage polarization, controlling the consequences of secondary injury after SCI, and assisting with function recovery. Herein, we review the mechanisms whereby MSCs affect macrophage-induced specific immune microenvironments, and discuss potential avenues of investigation for improving SCI treatment.

## Background

Spinal cord injury (SCI) is a serious complication of spine injury, which often leads to serious dysfunction of the limbs below the injured segment. It is one of the most common diseases leading to disability, and no effective treatments have been developed. Secondary injury is initiated shortly after the occurrence of primary SCI, resulting in irreversible damage to neurons and oligodendrocytes in the injured area. Inflammation caused by immune cells, such as macrophages, during the early and late stages of SCI is the main cause of secondary injury after SCI and prolongs the process of rehabilitation. In recent years, research on the application of MSCs for treating SCIs has gradually increased. Previous findings have shown that MSCs may elicit definite effects on macrophage polarization. Therefore, during clinical treatment, MSCs are expected to affect macrophage polarization, so as to change the inflammatory microenvironment of SCI and promote the recovery of neural functions.

In this review, we summarize the influence of macrophage polarization on the inflammatory microenvironment during SCI, the therapeutic effects of MSCs on SCI, and the regulatory mechanism of MSCs on macrophage polarization. We hope that the data highlighted in this review may provide guidance for stem cell-based therapy for SCI in the future.

### The pathophysiology of SCI

 SCI leads to motor, sensory, and autonomic nerve damage. In some cases, these defects may be caused by a loss of oligodendrocytes and demyelination in the remaining axons, resulting in slow or blocked conduction throughout the lesion [1]. SCI can be divided into primary and secondary SCI. The former refers to injury caused by the direct or indirect effects of an external force on the spinal cord. The latter refers to spinal cord edema caused by an external force, hematoma caused by small vessel hemorrhage in the spinal canal, compression fractures and ruptures of intervertebral disc tissues, and further damage to the spinal cord due to spinal cord compression. Due to its complex etiology, the pathogenesis of SCI is complex and includes oxidative stress, inflammatory responses, and glial scar formation. A key feature of SCI is that tissue destruction causes secondary damage and non-regressive inflammation, which aggravates the loss of function and impedes recovery. The early onset of inflammation after traumatic SCI emphasizes the importance of acute intervention after the initial trauma [2, 3]. Spinal neurons are sensitive to various injury-inducing factors, such as hypoxia and ischemia. Thus, when SCI interrupts downward projection, neurological dysfunction can result from subsequent denervation of lumbar motor neurons, neuronal cell death, and vascular injury [4–6].

Yet, despite all the damage caused, SCI is not untreatable. Maturation of the human immune system is related to recovery from injury-induced pathology and the recovery of neurological functions after SCI [7]. After the occurrence of SCI, immune cells can repair the tissue, close the wound, promote the removal of debris, inhibit the inflammatory response, and form a dense matrix by mobilizing immune cells and glial cells to form a protective barrier [8]. In addition, several prognostic indicators of SCI have been developed [9]. Among the various mechanisms leading to secondary SCI, inflammation is the most important because it directly or indirectly controls the sequelae after SCI. Inflammation in SCI can be divided into the following stages: neutrophil stimulation and invasion of the resident microglia on the second day, monocyte recruitment into the focus on the third through seventh days, and scar removal by anti-inflammatory macrophages and axon regeneration after 7 days [10].

Inflammation following SCI has both beneficial and destructive effects on tissues. Inflammation can lead to deterioration of the extracellular matrix and extensive cell damage. In the first week after SCI, early inflammatory events create an adverse microenvironment during the treatment of various SCIs, which creates obstacles to transplant-oriented treatment. During the acute and chronic SCI stages, systemic and local inflammatory reactions can lead to neurodegenerative events, forming cavities and glial scars in the parenchyma of the spinal cord, leading to neuron and glial cell death. Eliminating the pro-inflammatory environment of an injured spinal cord has become the main therapeutic approach for reducing secondary cell death and promoting neuronal regeneration. Data from recent studies have shown that inflammation is beneficial for functional recovery and neuronal regeneration [11, 12]. Inflammation in SCI plays an important role in clearing deteriorated and injured tissues, mediated by stimulated macrophages [13].

### **Influence of macrophage polarization on the inflammatory microenvironment during SCI**

After SCI, macrophages play important roles in mediating the inflammatory response during different periods.

The number of macrophages increased significantly at 3 and 7 days post-SCI injury, while macrophage-mediated inflammation peaked approximately seven days after injury [14]. Macrophages activated at the injury site can release inflammatory factors, chemokines, and mediators, and downregulate the expression of neurotrophic factors. Macrophages located in the lumbar spinal cord enhance the expression of various molecules, including C–C motif chemokine receptor 2 (CCR2), selectin L, and matrix metalloproteinase-9. The accumulation of these substances slows the recovery of patients’ functions [15]. Macrophages can produce growth factors that promote angiogenesis, stimulate fibroblast proliferation, and regulate connective tissue synthesis, which are the key elements of tissue repair. In addition to their roles as effectors of phagocytes and tissue repair, macrophages help trigger adaptive immunity [16]. However, after SCI, the role of macrophage activation remains controversial [17]. After SCI, the integrity of the damaged tissue and the recovery of nerve function can be improved by inhibiting or depleting macrophages [18]. However, injecting pre-activated monocytes into the spinal cord can promote axon growth and accelerate the improvement of motor function [19]. Thus, macrophage polarization plays important roles in SCI and damage repair mediated by the inflammatory response.

### Macrophage polarization

The term “polarization” refers to the phenomenon whereby macrophages exhibit different functional phenotypes in different microenvironments. This mainly includes “classically activated macrophages” (M1, pro-inflammatory) and “alternatively activated macrophages” (M2, anti-inflammatory). M1 and M2 macrophages can be produced following stimulation by different factors. They express different molecular markers and produce their own characteristic secretory factors, as shown in Table 1.

**Table 1:** Differences between M1 and M2 macrophages

Types	Inducing factor	Molecular marker	Characteristic cytokine
M1 macrophages	TH1 cytokines (such as IFN-γ, lipopolysaccharide, TNF-α)	iNOS, CD16/32, CD80, CD86, MHC II	IL-1β, IL-12, IL-6, IL-23, TNF-α, IFN-γ
M2 macrophages	TH2 cytokines (such as IL-4, IL-10, IL-13)	CD206, CD163, Arg1, Fizz1, YM1	IL-4, IL-10, IL-13, TGF-β

Within hours after SCI, macrophages (as first-response cells) become polarized into M1 macrophages after stimulation by helper T lymphocyte factors, such as interferon-γ (IFN-γ), lipopolysaccharide, and tumor necrosis factor (TNF)-α. The number of M1 macrophages peaks within one day after SCI. As a key signal-transduction factor, nuclear factor kappa B (NF-κB) plays an important role in M1 macrophage polarization. When NF-κB binds to the adenosine loop effector element binding protein (a regulatory transcription factor), it can stimulate gene transcription and cooperatively promote inflammatory gene transcription [20]. M1-type macrophages are regarded as harmful components during SCI. The induction of neuronal necrosis through various pathways and chondroitin sulfate proteoglycan expression inhibit neuron growth; pro-inflammatory M1-like macrophages produce pro-inflammatory cytokines and cytotoxic mediators induced by inflammatory factors, which simultaneously enhance their phagocytic and antigen-presentation abilities, and promote tissue destruction and microorganism killing, which in turn promotes the transformation of more macrophages to the M1 phenotype [21]. Activated M1-like macrophages express high levels of inducible nitric oxide synthase (iNOS), major histocompatibility complex II (MHC II), CD80, CD86, and CD16/CD32, and present antigens to T cells to activate and regulate innate and acquired immune responses [22]. The characteristic cytokines of M1 macrophages are interleukin (IL)-6, IL-12, and IL-23 [23]. M1 macrophages produce high levels of oxidative metabolites (such as nitric oxide and superoxide) and pro-inflammatory cytokines, which are essential for host defenses, but can also cause collateral damage to healthy cells and tissues [24]. M1 macrophages also produce other rejection-related factors, which induce axonal retraction after SCI [25]. Other evidence has shown that M1 macrophage polarization, triggered by IFN-γ and TNF-α, can decrease the phagocytic ability [26], which is very important for tissue repair after SCI.

M2-type macrophages are generated via induction with TH2 cytokines (such as IL-4, IL-10, and IL-13). The molecular markers of M2 macrophages are CD206, CD163, arginase 1 (ARG1), and found in inflammatory zone 1 (Fizz1) [27]. The characteristic cytokines are IL-10 and IL-4 [28]. During SCI, these macrophages help the body resist inflammation and promote tissue regeneration. They express IL-10 and transforming growth factor (TGF)-β and exhibit upregulated Arg1 expression. At a later stage, M2 macrophages can phagocytize scar tissue and myelin sheaths, which are harmful to nerve growth [29]. M2-like macrophages can inhibit the production of pro-inflammatory cytokines and affect macrophages by increasing MHC II expression. Transplanting M2 phenotypic macrophages or manipulating endogenous macrophages to acquire the M2 phenotype reduced spinal cord pathological injury, promoted regeneration, and improved functional recovery after SCI in rats [30]. Compared with wild-type mice, SCI mice lacking IL-10 displayed poor functional recovery [31]. Therefore, M2 macrophages have important research value for SCI treatment. However, excessive M2-like activation can promote the release of several growth factors, which in turn can promote the formation of fibrotic scars [32] and, thus, affect SCI recovery.

Given that most inflammatory events in the damaged central nervous system (CNS) occur after macrophage activation and migration, they may directly affect SCI repair, downstream inflammatory processes, secondary degeneration, and endogenous mechanisms [17]. The roles of polarized macrophages in the SCI microenvironment can be divided into advantageous and disadvantageous roles.

### The roles of polarized macrophages in the SCI microenvironment

#### Advantageous roles of polarized macrophages

Polarized macrophages can help remove harmful substances from injured sites in the spinal cord, assist in injury repair, and play active roles in nerve recovery.

Initially, M1 macrophages infiltrate sites of injury to repair wounds and remove bacteria, foreign bodies, and dead cells. IL-12 and IL-23 secretion by M1 macrophages can directly differentiate and expand anti-inflammatory TH1 and TH17 cells, which promotes processes related to mounting an inflammatory response and clearing invasive microorganisms [33]. Activated M1-like macrophages express high levels of MHC II and present antigens to T cells, which activates and regulates innate acquired immune responses [22]. The phagocytic and antigen-presentation abilities of M1-polarized macrophages are enhanced, which promotes the clearance of necrotic cells [34].

TGF-β and IL-10 are important anti-inflammatory factors expressed by M2 macrophages. These factors can promote the regeneration and neuroprotection of injured spinal cord tissue, as well as the renewal of damaged progenitor cells [13]. Accordingly, M2-like macrophages produce IL-10, the IL-1 receptor antagonist (IL-1Ra), and various chemokines, which induce tissue remodeling and promote the regeneration and growth of adult sensory nerve axons [24]. Some data have shown that transplanting M2 phenotypic macrophages may cause endogenous macrophages to acquire the M2 phenotype, mediate tissue remodeling, reduce pathological SCI, promote regeneration, and assist in functional recovery after SCI in rats. Therefore, M2 macrophages play active roles in repair and regeneration after SCI [30, 35].

#### Disadvantageous roles of polarized macrophages

Following SCI, necrotic cells release superoxide dismutase, which can induce macrophages to produce cytokines, such as IL-23, which accelerate nerve cell death [36]. In addition, M1 macrophages produce numerous inflammatory substances, such as reactive oxygen species (ROS) and reactive nitrogen species (RNS) through oxidative reactions, which inhibit cellular oxidative defenses and lead to further oxidative stress and SCI exacerbation [37].

M1 macrophages are neurotoxic [24]. They inhibit the establishment of the blood-brain barrier (BBB), aggravate lesions, promote cell death, and intensify immune responses. M1 macrophages also hinder the regeneration of the CNS [38]. Specifically, inflammatory factors (such as TNF-α, IL-β, ROS, RNS, prostaglandin 2, and other active substances) are secreted and released after M1 macrophage polarization, which damage neurons and glia, and even cause neuronal apoptosis [34, 39]. For example, cytotoxic ROS react with polyunsaturated fatty acids to cause lipid oxidation and degradation, which affect the fluidity and permeability of cell membranes and hinder cell metabolism and ion-channel exchange [40]. In addition, iNOS was highly expressed in lesions after SCI [41, 42]. Previous data also showed that ROS, RNS, and inflammatory substances produced by M1 macrophages can cause tissue damage [33].

#### Regulatory mechanism of macrophage polarization

**Fig. 1:**
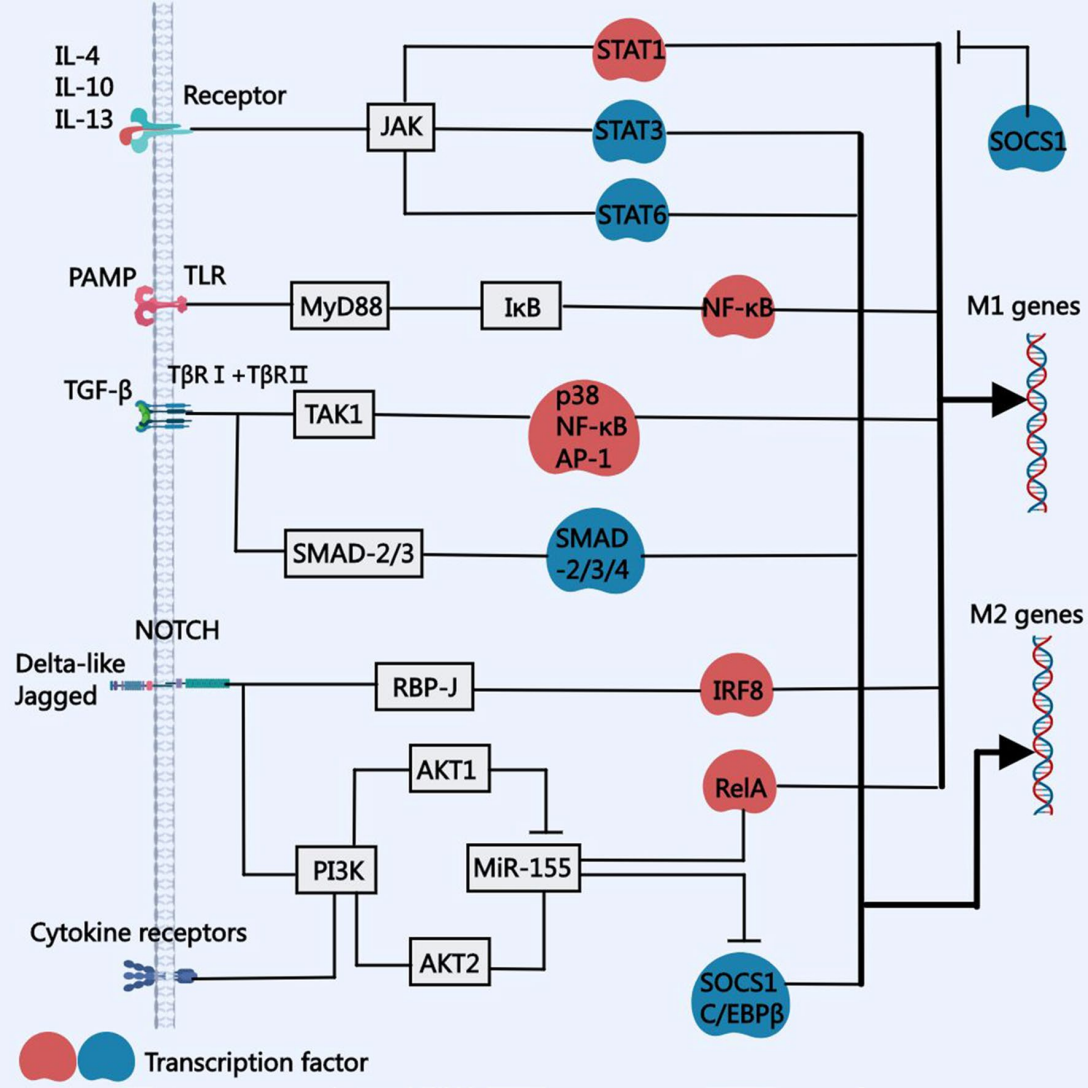
Regulatory mechanism of macrophage polarization. Macrophage polarization is a tightly controlled process involving a set of signaling pathways, including the PI3K–Akt, Notch, JAK–STAT, TGF-β, and TLR4–NF-κB signaling pathways. AKT, protein kinase B; JAK: Janus kinase; JNK: c-Jun N-terminal kinase; NF-κB, nuclear factor kappa-B; PI3K, phosphoinositide 3-kinase; STAT, signal transducer and activator of transcription; TGF-β, transforming growth factor beta; TLR, Toll-like receptor

Macrophage polarization is regulated by various signaling molecules and their related pathways. At present, the main signaling pathways include the phosphatidylinositol-3-kinase (PI3K)–protein kinase B (Akt) signaling pathway, the Notch-signaling pathway, the Janus kinase (JAK)–signal transducer and activator of transcription (STAT)-signaling pathway, the TGF-β-signaling pathway, and the Toll-like receptor (TLR) 4–NF-κB-signaling pathway (Fig. 1).

#### PI3K–Akt-signaling pathway

Regarding the PI3K–Akt pathway, it is necessary to first understand the main regulatory factors, namely PI3K and Akt. PI3K is an intracellular phosphatidylinositol kinase that is activated by cytokine receptors and TLRs [43]. The AKT family consists of three serine–threonine kinases, namely Akt1, Akt2, and Akt3 [44]. PI3K can produce phosphatidylinositol (3, 4, 5)-triphosphate and activate protein kinase Akt, which induces macrophage polarization by regulating microRNA-155 (miR-155) expression. Specifically, Akt1 promotes the formation of the M2 phenotype, and Akt2 promotes the formation of the M1 phenotype [45]. In mechanistic terms, Akt2 enhances miR-155 expression at the transcriptional level, which leads to primary (pri)-miRNA formation, subsequent processing to pre-miRNA, followed by the production of mature miRNA. Following increased pri-miR-155 production, miR-155 expression increases, which interferes with the cAMP-response element binding protein (CREB)–CCAAT-enhancer-binding protein β (C/EBPβ) cascade, weakens M2-specific gene upregulation, and simultaneously downregulates C/EBPβ. C/EBPβ is a member of the C/EBP basic-region leucine zipper protein family, which can transcriptionally activate the IL-10 and Arg1 promoters. The IL-10 and Arg1 promoters induce M1 macrophage polarization [46]. In addition, increased miR-155 expression also inhibited synthesis of the cytokine signal-transduction inhibitor 1 (SOCS1) protein, which is an important regulator of M1/M2 macrophage polarization, which promoted formation of the M1 phenotype [47, 48]. In contrast, Akt1 inhibits miR-155 expression, thus increasing the expression of C/EBPβ and genes related to the M2 phenotype (ARG1 and IL-4), in turn promoting M2 macrophage polarization [46].

#### Notch homolog 1, translocation‐associated (Notch)-signaling pathway

Four different Notch receptors (Notch1, Notch2, Notch3, and Notch4) participate in the mammalian Notch-signaling pathway and are expressed in various tissues and organs. Notch receptors are comprised of a single transmembrane domain with functional extracellular, transmembrane, and intracellular domains. Notch ligands can be divided into Delta-like (DLL1, DLL3, and DLL4) and Jagged (JAG1 and JAG2) families [49]. These ligands bind to the same or different Notch receptors and activate them, which stimulates the Notch-signaling pathway. The Notch-signaling pathway triggers many regulatory effects in terms of macrophage polarization, which are described below:

① Mechanistically, the Notch-signaling pathway mainly activates the recombination signal binding protein for immunoglobulin kappa J region (RBP-J) transcription factor and interferon regulatory factor (IRF)8, which reprograms the M1 and M2 phenotypes of macrophages through the PI3K pathway. Macrophages expressing DLL4 receptors can induce Notch proteolysis, resulting in increased IL-12 gene activity, which in turn induces macrophages to acquire the M1 phenotype [50].

② RBP-J is an important transcription factor in the Notch-signaling pathway, and Notch1 activation can promote RBP-J production. RBP-J increases IRF8 expression in macrophages. Importantly, IRF8 not only participates in the Notch-signaling pathway, but also activates the TLR-4-signaling pathway to produce pro-inflammatory M1 cytokines [51]. Compared with non-reprogrammed macrophages, with an increased IRF8 content, the combined effects of ligands and TLR-4 on reprogrammed macrophages can lead to more significant inflammatory reactions, which is a pro-inflammatory feature of reprogrammed macrophages [52].

③ The Notch1-signaling pathway can also promote formation of the pathogenic M2 phenotype [53]. In this case, the Notch1-signaling pathway translates signals through the PI3K pathway, activates genes related to the M2 phenotype, and reprograms macrophages to acquire the M2 phenotype [54].

④ The STAT gene is also important for the Notch-signaling pathway. The STAT gene is encoded on chromosome 17 and is a kind of proto-oncogene. SOCS3 is an M2 transcription factor that inhibits expression of the STAT3 gene. STAT expression can lead to increased Notch–RBP-J pathway-dependent reprogramming of macrophages to the M1 phenotype. STAT function represents the mechanism between increased pro-inflammatory cytokine production and decreased anti-inflammatory cytokine production [53].

#### JAK–STAT-signaling pathway

JAKs are non-receptor tyrosine protein kinases that are activated by various cytokines. Downstream target genes are activated by STATs, and the corresponding expressed proteins play regulatory roles. The JAK–STAT pathway is mainly composed of three parts: tyrosine kinase-related receptors, JAKs, and STATs. Four types of JAKs have been discovered, namely JAK1–3 and tyrosine kinase 2 (TYK2). In addition, six types of STATs are known (STAT1–6). The SH2 structural region of STAT proteins is identical to the corresponding core sequence in JAK proteins, which is responsible for recognizing specific JAKs. The basic signal-transduction pathway involves cytokine–receptor binding on the cell surface, receptor dimerization, and JAK polymerization and phosphorylation. Activated JAKs can bind the SH2 domains of STATs, become activated after STAT phosphorylation, and finally enter the nucleus in the form of a homodimer or a heterodimer to promote the transcription of target genes [55]. The JAK–STAT pathway regulates many effects in terms of macrophage polarization, as described below:

① IFN-γ induces STAT1 activation through the JAK–STAT pathway. STAT1 then acts as a homodimer to bind the cis element (known as the IFN-γ-activation site) in the promoters of genes related to M1 polarization, resulting in increased production of pro-inflammatory cytokines and acquisition of the M1 phenotype [56]. The IFN-γ–JAK–STAT1 pathway is controlled by IRF4 and IRF5 [57]. They have opposite effects in terms of the IFN-γ–JAK–STAT1 pathway and macrophage phenotype. IRF5 can be activated by pro-inflammatory factors, whereas IRF4 can be activated by anti-inflammatory factors [58]. IRF5 promotes the production of IL-12, an M1 cytokine dependent on the IFN-γ–JAK–STAT1 pathway, whereas IRF4 inhibits the effects of IRF5 [58].

② IL-4, IL-13, and IL-10 polarize macrophages to the M2 phenotype through the JAK–STAT-signaling pathway. IL-4 binds to its receptor to activate JAK. Then, two transcription factors of genes related to the M2 phenotype (STAT3 and STAT6) are phosphorylated and activated [59]. In addition, IL-4 can also induce c-Myc gene expression, which in turn increases the expression of M2-phenotype genes, such as scavenger receptor class B1 and mannose receptor, C type 1, as well as the activity of STAT6 and peroxisome proliferator-activated receptor (PPAR)-γ [60].

③ IL-13 binds to receptors to activate the JAK1, JAK2, and TYK2 kinases, and then activates STAT1, STAT3, and STAT6. STAT3 and STAT6 activate the expression of M2-phenotype genes, such as the mannose receptor, Fizz1, chitinase-like protein 3 (YM1), and anti-inflammatory cytokines, whereas STAT1 activates pro-inflammatory cytokines. The anti-inflammatory response of macrophages induced by IL-13 may reflect the fact that STAT3 and STAT6 are more extensively activated than STAT1 [61].

④ IL-10 binds to receptors to activate JAK1 and STAT3, resulting in activation of M2-phenotype genes, such as TGF- β and IL-10 [61].

⑤ The SOCS1 and SOCS3 proteins are two important regulators of the JAK–STAT-signaling pathway during M1/M2 macrophage polarization [47]. IL-4 activates SOCS1 synthesis and blocks STAT1 production, thus preventing formation of the M1 phenotype. IFN-γ and TLR4 ligands activate SOCS3 synthesis, which blocks STAT3, thus preventing the formation of the M2 phenotype [62]. In addition, SOCS1 activated the M2 phenotype-related transcription factor STAT6, while SOCS3 activated M1 phenotype-related transcription factor STAT1. These interactions between SOCS and STAT further explain the relationship between the increase of pro-inflammatory cytokines and the decrease of anti-inflammatory cytokines produced by macrophages during polarization to the M1 phenotype [44].

#### TGF-β-signaling pathway

The TGF-β protein family includes TGF-β1, TGF-β2, TGF-β3, activin, and several growth factors. Macrophages mainly produce TGF-β1. The TGF-β receptor is composed of two type-I transmembrane subunits (TβRI) and two type-II subunits (TβRII) with a cytoplasmic kinase domain. After TGF-β binds with its receptor, TβRII is auto-phosphorylated and then phosphorylates TβRI. Next, the TβRI cytoplasmic kinase domain binds to and phosphorylates (activates) SMAD2 and SMAD3. Activated SMAD2 and SMAD3 bind to SMAD4, and the resulting ternary complex translocates into the nucleus, which upregulates the activities of the M2 phenotype-related genes ARG1 and MGL2, and reprograms macrophages to acquire the M2 phenotype [63].

In addition, TGF-β-dependent reprogramming of the M2 phenotype is related to SMAD7. SMAD7 can bind to TβRI, which prevents SMAD2 and SMAD3 phosphorylation, and can direct both SMADs to the proteasome for degradation. The proinflammatory factors IFN-γ and TNF-α can upregulate SMAD7 expression, thus inhibiting the TGF-β–SMAD pathway and reducing the production of anti-inflammatory factors. This mechanism can help explain the relationship between increased production of pro-inflammatory factors and decreased production of anti-inflammatory factors [64].

In addition to the SMAD-dependent TGF-β-signaling pathway, TGF-β can also activate the SMAD-independent TGF-β-signaling pathway. In the SMAD-independent pathway, the TGF-β-activated kinase protein 1 (TAK1) transduces signals from TGF-β to several downstream signal cascades, including the transcription factors JNK, p38, and NF-κB [44]. These SMAD-independent pathways can reprogram macrophages to acquire the M1 phenotype. When the SMAD-dependent pathway is blocked, the effect of the SMAD-independent pathway is more pronounced [65].

#### TLR4–NF-κB-signaling pathway

TLRs belong to the pattern-recognition receptor transmembrane family, and six TLRs (TLR1, TLR2, TLR4, TLR5, TLR6, and TLR10) have been identified on the surface of macrophages. TLRs can identify specific pathogen-associated molecular patterns (PAMPs) on microbial molecules. PAMP binding to TLRs triggers signal cascades, which induce macrophage reprogramming into the M1 phenotype [66]. When PAMPs bind to TLRs, they dimerize, which activates myeloid differentiation primary response protein 88 (MyD88) [66]. MyD88 combines with members of the IL-1R related kinase (IRAK) family to form the Myddosome complex [67].

IRAK is phosphorylated in the Myddosome complex, phosphorylated IRAK attracts tumor necrosis factor receptor related factor-6 (TRAF6) to the cell membrane, and TRAF6 can attract the TAK1 complex [68].

TRAF6 attracts the TAK1 complex, which causes autophosphorylation and activation of TAK1 kinase, which in turn activates the IκB kinase (IKK) complex [69]. IκB functions as an inhibitory subunit related to the NF-κB transcription factor in the cytoplasm of macrophages. IκB can be phosphorylated by IKK, which leads to its degradation in proteasomes. Free NF-κB is transported to the nucleus, where it activates genes related to inflammation, immune responses, and cell growth [69].

There are five proteins in the NF-κB family in mammals, namely RelA (p65), RelB, c-Rel, NF-κB1 (p50), and NF-κB2 (p52). Proteins belonging to the p65, RelB, and c-Rel families can induce macrophage reprogramming into the M1 phenotype, whereas proteins belonging to the p50 and p52 Relish families have no such effect [70]. Pro-inflammatory cytokines produced by the NF-κB-dependent pathway can repeatedly activate the NF-κB-dependent pathway and form a positive-feedback loop, such that macrophages rapidly acquire the M1 phenotype. NF-κB can also activate the IκB gene, limit excessive nuclear translocation of NF-κB, and act as a negative-feedback mechanism to prevent excessive inflammation [71].

In summary, macrophage polarization is highly regulated by various molecules and signaling pathways. Defining the key pathways and regulatory factors that regulate macrophage polarization is key for promoting the polarization of pro-inflammatory M1 macrophages into anti-inflammatory M2 macrophages and, thus, improving the unfavorable inflammatory microenvironment after SCI and promoting SCI repair. MSCs, as a source of adult stem cells, are easy to obtain. The regulation and mechanism of MSCs on SCI repair and macrophage polarization have been expounded upon, and such insights have important guiding significance for applying MSCs to autologous stem cell therapy after SCI.

### MSCs alleviate SCI by inducing macrophage/microglial cell polarization

#### *MSC transplantation—an effective way to repair SCI*

SCI can result in severe disabilities associated with motor, sensory, and autonomic dysfunctions. Stem cell transplantation is considered a potential therapy for stimulating neural plasticity and nerve regeneration after SCI [72]. MSC transplantation is a promising approach for treating SCI. It can be widely used in clinical practice because, compared with other stem cells, such as embryonic stem cells and induced pluripotent stem cells, MSCs are more easily obtained, and few ethical and safety concerns are associated with autologous transplantation [73]. MSC-derived neural network tissue transplanted in this way can be used as a “neuronal relay” of structure and function to restore the motor functions of paralyzed limbs of organisms with complete SCI [74]. In addition, transplanted MSCs can not only provide tissue replacement, but can also release the nutrients and matrix components needed for tissue regeneration. Transplanted MSCs also exert anti-inflammatory effects by downregulating pro-inflammatory factors [75]. Evaluating histopathological changes, proinflammatory cytokine levels, and locomotor functions, and verifying the effects of exogenous bone marrow stromal cell (BMSC) lines and BMSCs on animals with SCI have revealed that intravenous BMSCs have good therapeutic effects [76]. In addition, the current main functions of cell therapy include restoration of sphincter dysfunction and relief from neuropathic pain, which are very safe and effective for use in treating patients with SCI [77].

At present, MSC-based SCI treatment mainly includes intravenous injection of MSCs, intravenous injection of BMSC-derived exosomes, and transplantation of BMSCs + scaffold materials.

Intravenous infusion of BMSCs promoted functional recovery after contusive SCI in non-immunosuppressed animals following local treatment [78], which can provide neuroprotection, stabilize the blood–spinal cord barrier (BSCB), regenerate the myelin sheath, and germinate axons. Compared with other cell types, BMSCs are ideal transplantable cells and mainly regulate the SCI cascade through a paracrine mechanism. After BMSCs were infused, the myelin sheath regenerated widely around the focus center, and the sprouting of corticospinal tract and serotonergic fibers increased. In addition, systemic infusion of BMSCs can lead to functional improvement related to structural changes in chronically injured spinal cords, including BSCB stabilization, axonal germination and regeneration, and remyelination [79].

In addition, transcranial magnetic stimulation (alone or together with human umbilical cord blood MSC transplantation) alleviated neural stem cell apoptosis and motor dysfunction induced by SCI [80]. Ischemic SCI (ISCI) is a devastating complication of aortic surgery with few preventive strategies. Intravenous BMSC infusion has been shown to provide functional improvement for patients with ISCI. Potential therapeutic mechanisms include the neuroprotection of white and gray matter and damaged spinal cord, reduction of axon loss or degeneration, and reduction of BSCB damage in the damaged spinal cord. Therefore, BMSC therapy may have therapeutic value in ISCI [81].

SCI leads to a strong inflammatory reaction, which greatly affects the functions of stem cells [82]. Whether scaffolds can be used as adjuvant therapies for MSC transplantation in SCI has long been controversial. Therefore, a meta-analysis of preclinical evidence was conducted to evaluate the effectiveness of stent + MSC transplantation in improving SCI motor dysfunction, compared with stent or MSC treatment alone. The results showed that, in the acute-injury stage, after SCI, stent + MSC treatment was more effective in improving motor functions than using a stent and MSCs alone [83].

#### The mechanism whereby MSCs regulate macrophage polarization in the context of SCI

The results of several studies have shown that MSC transplantation after SCI can promote the polarization of macrophages/microglia from the M1 phenotype to the M2 phenotype, improve the inflammatory environment, and promote recovery after SCI [84, 85]. It was reported that TNF-α produced by the spleen plays an important role in activating transplanted MSCs, suggesting that the spleen is involved in MSC-mediated effects [86]. Furthermore, compared with allogeneic MSCs, syngeneic MSCs have superior therapeutic potential [87]. Combined treatment with biomaterial and MSCs may provide a promising therapeutic treatment for SCI. For example, Zhan et al. showed that MSCs loaded on a nerve-guided collagen scaffold could better promote M2 macrophage polarization [88]. MSCs combined with a three-dimensional biomimetic hydrogel could deliver cytokines to the SCI site, thereby increasing the M2 macrophage population and promoting a pro-regenerative environment [89].

**Fig. 2:**
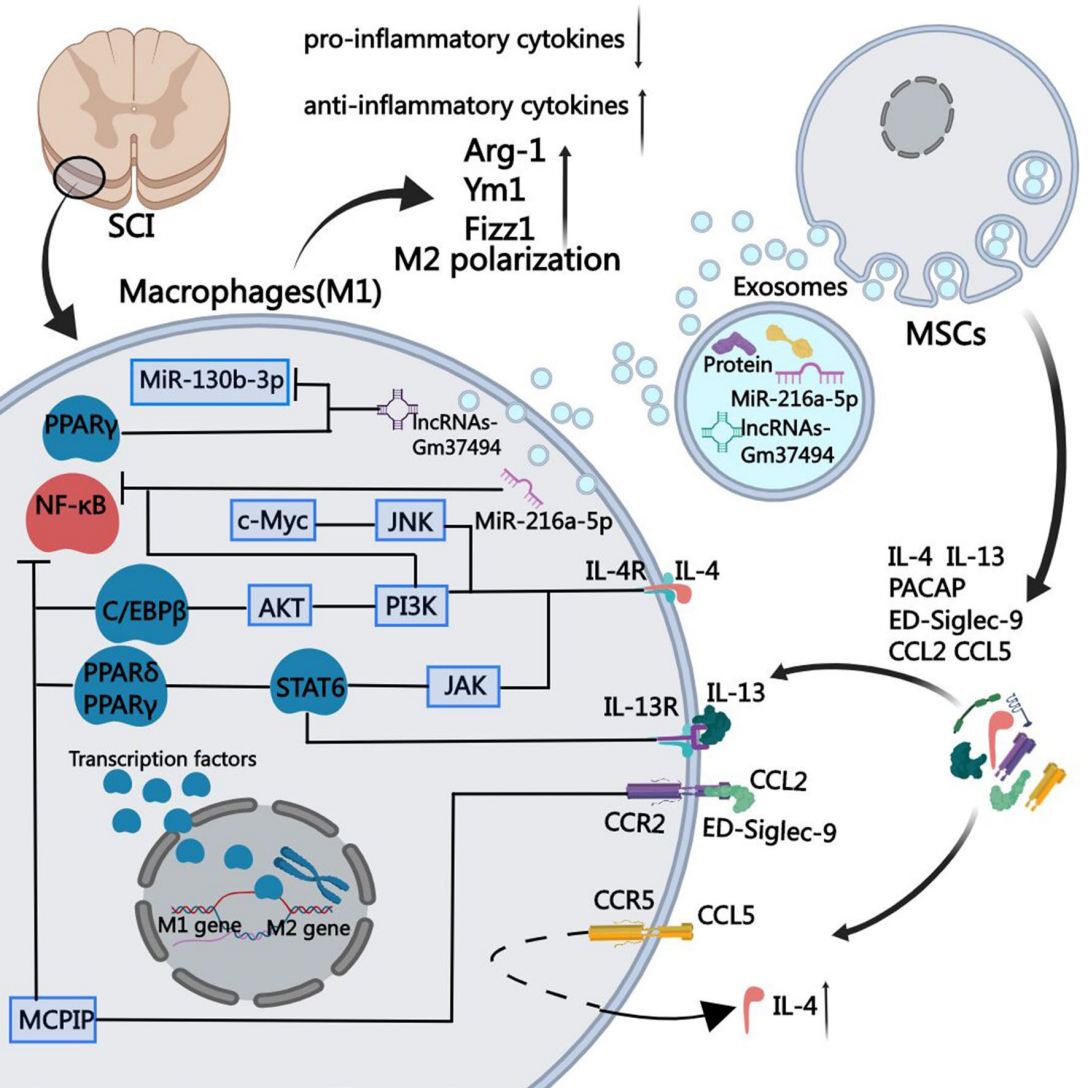
Mechanism by which MSCs regulate macrophage polarization in the context of SCI. MSCs can promote the polarization of macrophages at the site of SCI by secreting soluble proteins including IL-4, IL-13, PACAP, CCL2, CCL5, and ED-Siglec-9. IL-4 can activate the JNK, JAK/STAT6, and PI3K signal pathways through IL-4R, and ultimately promote the expression of M2 related genes. In turn, IL-13 activates IL-10R, thereby participating in the process by acting on STAT6. In addition, CCL5 can induce high levels of IL-4 by acting on CCL5R, thereby promoting M2 polarization of macrophages. CCL2 can induce the expression of MCPIP through CCR2, thereby activating C/EBPβ, PPARγ, and inhibiting NF-κB to exert anti-inflammatory effects. ED-Siglec-9 also works synergistically with CCR2. Regarding PACAP, its mechanism of action is not yet clear. Furthermore, MSCs-exo can also play a similar role, by carrying MiR-216a-5p and lncRNAs Gm37494, which will inhibit TLR4/NF-κB and activate PI3K/AKT signaling pathway, and downregulate the expression of MiR-130b-3p and upregulate the expression of PPARγ, respectively, hence, inhibiting M1 and enhancing M2 marker expression. Lastly, MSCs can improve the inflammatory environment of the SCI site and relieve its manifestations. AKT, protein kinase B; CCL, chemokine (C-C motif) ligand; CCR, chemokine (C-C motif) receptor; C/EBPβ, CCAAT/enhancer-binding protein beta; ED-Siglec-9, ectodomain of sialic acid-binding Ig-like lectin-9; IL, interleukin; JAK, Janus kinase; JNK, c-Jun N-terminal kinase; lncRNAs, long non-coding RNAs; MCPIP, monocyte chemoattractant protein-1-induced protein.; MSCs, mesenchymal stem cells; NF-κB, nuclear factor kappa-B; PACAP, pituitary adenylate cyclase-activating polypeptide; PI3K, phosphoinositide 3-kinase; PPARβ, peroxisome-proliferator-activated receptor β; SCI, spinal cord injury; TLR, Toll-like receptor

In terms of adjustment methods, MSCs can induce macrophage polarization by secreting soluble factors or exosomes in the context of SCI (Fig. 2).

#### Secretion of soluble factors

MSCs can regulate macrophage polarization by secreting soluble proteins, including ILs and chemokines, which ultimately relieve SCI.

Multiple findings have shown that ILs play important roles in macrophage polarization. IL-4 and IL-13 are classic inducers of the M2 macrophage phenotype [90, 91]. IL-4 induces c-Jun N-terminal kinase (JNK) in macrophages, leading to subsequent downstream transcription of c-Myc in conjunction with IL-4Rα, as well as increased expression of the M2 markers, Arg1 and Mrc1 [92, 93]. In addition, IL-4 upregulates the expression of M2 macrophage genes (such as Arg-1 and YM1) by activating the IL4-Rα–JAK–STAT6 and PI3K pathways, which facilitate M2 macrophage polarization. PPARγ and PPARδ also participate in the process as downstream signals of STAT6 [94, 95]. IL-13 acts on a complex receptor composed of IL-4R and IL-13Rα, and upregulates M2 macrophage markers by activating STAT6 to promote macrophage polarization [95]. Researchers transplanted MSCs genetically engineered to secrete IL-13 into mice with SCI and observed a significant increase in M2 macrophages in the transplanted areas. It is worth noting that the majority of these are peripherally derived macrophages [96]. Furthermore, using an anti-IL-7Rα monoclonal antibody to block IL-7 signal transduction after SCI can promote M2 macrophage induction [97]. Another study provided evidence that transplanted human umbilical cord MSCs can promote M2 macrophage polarization by decreasing IL-7 expression, thereby alleviating SCI [98].

Research conducted by Tomomi et al. showed that implanting human MSCs into mice with SCI induced M2 macrophages through a mechanism related to IL-4 upregulation. The neuropeptide, pituitary adenylate cyclase-activating polypeptide, induced by the microenvironment also participates in crosstalk between MSCs and macrophages and enhances macrophage polarization, although the specific mechanism involved remains unclear [99].

Chemokines promote the migration of immune cells and participate in inflammatory reactions, which can be used to research communications between MSCs and macrophages [100]. Chemokine (C-C motif) ligand 2 (CCL2), also known as monocyte chemotactic/chemoattractant protein 1 (MCP1), is an inflammatory chemokine produced by monocytes [101]. CCL2 functions by binding to CCR2 and initiating intracellular signal transduction [102]. Elena et al. showed that CCL2 and CCR2 are differentially expressed in macrophages. Compared with M1 macrophages, M2 macrophages express higher levels of CCL2; however, CCR2 surface expression is only observed with M1 macrophages. For this reason, only M1 macrophages can respond to CCL2 stimulation [103]. Previous findings have shown that CCL2 can not only recruit peripheral pro-inflammatory M1 macrophages, but can also promote M2 macrophage polarization [104, 105]. The specific mechanism involves the targeted binding of CCL2 and CCR2, and a series of downstream changes, including increased activation of p38, ERK1/2, MSK1/2, HSP27, JNK, and STAT5a/b [103], increased activation of the p42 and 44 mitogen-activated protein kinases [103, 106], and upregulation of MCP-1-induced protein (MCPIP), which inhibits NF-κB activation and induces C/EBPβ and PPARγ [107, 108]. Researchers have used biomimetic hydrogel scaffolds encapsulated with MSCs to treat SCI in mice. The results showed that CCL2 secreted by MSCs can be effectively transferred to the diseased spinal cord, leading to downregulated expression of M1 markers (TNF-α and IL-1β) and upregulated expression of M2 markers (YM1 [also known as Chil3], is a member of the chitinase-like protein family) and Arg-1, which promoted peripheral macrophage M2 polarization [109]. In another study, MCP-1 and a previously unrecognized inducer of M2 macrophages were analyzed in the medium of MSCs, which revealed secretion of the ectodomain of sialic acid-binding Ig-like lectin-9 and synergistic induction of M2-like macrophages through CCR2 [105]. In addition, research conducted using a SCI mouse model showed that transplanted MSCs can cause neuronal cells to secrete CCL2 and induce CCR2 expression in granulocytes. Through targeted CCL2–CCR2 binding, MSCs can increase macrophage expression of the zinc finger CCCH-type containing 12A protein (encoded by MCPIP and involved in M2 polarization), which can inhibit NF-κB signaling and ultimately induce M2 polarization and promote recovery from SCI in rats [110]. Chemokine (C–C motif) ligand 5 (CCL5), also known as RANTES, is one of the members of the mammalian chemokine system [111]. CCL5 is usually studied as a marker of M1 macrophage polarization because it acts on multiple receptors, both typical (CCR1, CCR3, CCR5, and CCR4) and atypical (non-signaling receptors like ACKR1, ACKR2, and CCRL2) [112, 113]. The above research also demonstrated that transplanted MSCs caused neuronal cells to secrete CCL5, which bound CCR5 on the surface of macrophages. IL-4 is the main regulator of macrophage polarization [91]. CCL5–CCR5 binding can promote M2 polarization by inducing high levels of IL-4 and upregulating the expression of Arg-1 and YM1, which are markers of M2 macrophages. In addition, CCL5–CCR5 binding can promote recovery from SCI [110].

#### Exosomes

Exosomes are membrane-like lipid vesicles (30–100 nm in size), which contain mRNAs, miRNAs, long noncoding RNAs (lncRNAs), and proteins, and play important roles in information transfer between cells [114, 115]. Exosomes can bind to target cells and release their contents through specific cell-surface ligands, thereby regulating specific biological functions, such as immune responses and angiogenesis [116, 117]. Data from previous studies have shown that MSCs can alleviate myocardial ischemia-reperfusion injury, myocardial infarction, sepsis, stroke, and neurological injury by secreting exosomes to induce macrophage polarization [118–121].

Regarding SCI, previous research showed that intravenous MSC-derived exosomes (MSC-exos) can specifically target M2-type macrophages at the SCI site [78]. Zhao et al. showed that MSC-exos mainly gather at the SCI site and bind to microglia (to inhibit complement release) and SCI-activated NF-κB (to inhibit inflammatory damage) [122]. These findings suggest that MSC-exos may mediate some of the functional roles of MSCs in SCI.

MSC-exos also alleviate SCI by participating in macrophage polarization. In an SCI model, MSC-exos promoted functional recovery by inducing the mRNA expression of M2 macrophage markers, including CD206, IL-10, and Arg-1, and facilitated bone marrow-derived macrophage polarization from an M1 to an M2 phenotype. In addition, MSC-exos also decreased the levels of pro-inflammatory cytokines (TNF-α, IL-6, granulocyte colony-stimulating factor, IFN-γ, MCP-1, and macrophage inflammatory protein-1α), while increasing the levels of the anti-inflammatory cytokines, IL-4 and IL-10 [123].

Oxygen concentration plays an important role in the processes of MSC proliferation, differentiation, and self-renewal [124]. However, the oxygen concentration under in vitro culture conditions (21%) is much higher than that in the body under physiological conditions (≤ 2–8%). Hypoxic pretreatment of MSCs (to simulate the hypoxic environment in the body) can significantly improve their biological functions and activities, thereby improving the efficacy of treatment in various disease models [125, 126]. Correspondingly, exosomes secreted by MSCs with hypoxic pretreatment showed better repair effects than the exosomes secreted by normal MSCs, suggesting that hypoxia preconditioning is a promising and effective approach for improving the efficacy of MSC-exos [127]. Liu et al. showed that hypoxia-preconditioned MSC-exos were rich in miR-216a-5p, which was transferred to microglia and targeted expression of the TLR4 gene, thereby inhibiting the TLR4–NF-κB pathway and activating the PI3K–AKT pathway, eventually promoting polarization of microglia to alleviate SCI [128]. Shao et al. reported that exosomes secreted by hypoxia-pretreated human amnion-derived MSCs (HExos) showed inhibited expression of inflammatory factors and increased polarization of microglia from M1 to M2, which promoted functional recovery after SCI. Importantly, high-throughput sequencing analysis revealed high expression of the lncRNA, Gm37494, in HExos, which can downregulate miR-130b-3p expression and upregulate PPARγ expression. The former can promote the expression of inflammatory factors, and the latter can inhibit the NF-κB light-chain enhancer (related to STAT activity) to inhibit NF-κB and the expression of M1 markers [129, 130].

## Summary and discussion

A series of pathophysiological changes occur after SCI. The primary injury is related to the destruction of axons and neurons. The secondary injury is caused by nerve inflammation, which directly or indirectly controls the sequelae of SCI and can lead to morphological edema, cavitation, and reactive glial hyperplasia. SCI treatment is challenging because it can lead to many irreversible pathological reactions. Given that the immune response in SCI is a “double-edged sword,” beneficial aspects should be promoted during treatment, rather than completely inhibiting inflammation. The pro-inflammatory and anti-inflammatory effects of macrophages during different stages of SCI are important causes of symptoms at different periods. The direction of polarization can differ in macrophages, and many pathways and cytokines are involved in regulating polarization. The representative pathways are the PI3K–Akt-signaling pathway, the Notch-signal pathway, the JAK–STAT-signal pathway, the TGF-β-signaling pathway, and the TLR4–NF-κB-signaling pathway. MSCs have great potential for spinal cord repair and represent promising candidates for long-term treatment of secondary SCI, caused by neuroinflammation.

Although many studies involving SCI treatment have been based on BMSCs, some problems remain. For example, MSCs transplanted into the spinal cord have a low survival rate and may differentiate into other types of cells, such as osteoblasts, which limits the therapeutic effect of BMSCs. BMSCs play an important therapeutic role through exocrine secretions, and the direct application of exocrine secretions as a therapeutic agent is one research direction [131]. However, the lack of exocrine-production capacity and its low targeting are the main factors that limit this strategy at present. Moreover, applying conditioned medium from BMSCs represents an alternative method for MSC transplantation in SCI treatment, but this method is still under development [132]. Many drugs and treatments have proven effective in experimental studies, but their actual clinical effects are unknown. Beyond macrophages, T cell reduction can improve the recovery of the spinal cord structure and limb function after SCI, but the related mechanisms remain unclear [133–135]. The beneficial and detrimental effects of B cells before and after SCI damage to the BBB remains controversial [136]. In future research, dynamic changes in the immune system after SCI should be explained. Further exploration and experiments are needed to coordinate the interaction between different cells and improve the efficacy of nerve injury treatment. In addition to exploring the strategy of directly applying MSCs, treatment with BMSCs should also be studied in terms of specific functional exocrine secretions or conditioned MSC medium, in combination with other clinical strategies to lay the foundation for improved practical applications.

## Data Availability

The datasets used and/or analyzed during the current study are available from the corresponding author on reasonable request.
